# Investigation of Antiarthritic Potential of *Plumeria alba* L. Leaves in Acute and Chronic Models of Arthritis

**DOI:** 10.1155/2014/474616

**Published:** 2014-06-15

**Authors:** Manjusha Choudhary, Vipin Kumar, Pankaj Gupta, Surender Singh

**Affiliations:** ^1^Institute of Pharmaceutical Sciences, Kurukshetra University, Kurukshetra, Haryana 136119, India; ^2^Department of Pharmacy, School of Chemical Science and Pharmacy, Central University of Rajasthan, Ajmer 305801, India; ^3^Dabur Research and Development Centre, Ghaziabad 201002, India; ^4^All India Institute of Medical Sciences, New Delhi 110029, India

## Abstract

*Aim*. The present investigation was designed to evaluate antiarthritic potential of fractions of hydroalcoholic extract from leaves of *P. alba*. *Materials and Methods*. *Plumeria alba* L. leaves were extracted with hydroalcohol (30 : 70) to obtain hydroalcoholic extract of *P. alba*. This extract was further fractionated with solvents ethyl acetate and n-butanol to obtain EAPA and BPA, respectively. These fractions were tested against formaldehyde and Freund's complete adjuvant (FCA) induced arthritis. Arthritis assessment, paw volume, body weight, motor incoordination, and nociceptive threshold were measured. On day 21, the animals were sacrificed and histopathology was done. *Results*. The 100 and 200 mg/kg doses of EAPA and BPA caused a significant (*P* ≤ 0.05–0.01) reduction in paw swelling in both models. Erythrocyte sedimentation rate (ESR) and spleen weight decreased significantly (*P* < 0.01) in arthritic rats treated with extracts. There was significant (*P* < 0.05) improvement in thymus weight in EAPA treated rats whereas significant (*P* < 0.01) improvement was also seen in haemoglobin level (Hb) in diclofenac treated group. Motor incoordination and nociceptive threshold were also significantly (*P* ≤ 0.05–0.01) improved. *Conclusion*. The present study suggests that *Plumeria alba* L. has protective activity against arthritis and supports the traditional use of *P. alba* for rheumatism and other inflammatory diseases.

## 1. Introduction

Inflammatory diseases like rheumatism are a major and worldwide problem. Rheumatic arthritis (RA) is a chronic, inflammatory systemic autoimmune disease that affects mainly Western countries and is more common in females than in males [1]. Although the aetiology underlying RA remains unknown, it is clear that inflammatory cytokine circuits are established in the synovial cells lining the joint in genetically susceptible individuals. The inflammatory process in RA initially affects the synovial membrane lining but can affect other organs also. The inflamed synovium leads to the aggressive cartilage destruction and progressive bony erosions. The disease is often progressive and results in pain, stiffness, and swelling of joints. In late stage deformity and ankylosis develop [2].

The modern drugs both steroidal and nonsteroidal anti-inflammatory drugs (NSAIDS) like etoricoxib and disease modifying antirheumatic drugs (DMARDSs) like methotrexate sulphasalazine, hydroxychloroquine, and leflunomide are used for the amelioration of the symptoms of the disease; however, they offer only temporary relief and also produce adverse effects [3]. Because of this reason, patients suffering from chronic musculoskeletal disorders are likely to seek alternative methods for symptomatic relief and are amongst the highest users of complementary and alternative medicine [4, 5].

Genus* Plumeria *L. (Apocynaceae) originated from Central America and its different species are now distributed in the warmer regions of the world including India. The genus is represented by topical trees or shrubs, frequently cultivated as ornamental and medicinal plants [6]. In Ayurvedic system of Indian medicine, the plants of* Plumeria *species are widely used in gastric ulcer and rubefacient in rheumatism, asthma, piles, gonorrhoea, blood disorders, and tumours [7].

*Plumeria alba *L., commonly called white Champa, is 4.5 m high, cultivated in Indian gardens and popularly called “peru” in Tamil [8]. The plant is mainly grown for its ornamental and fragrant flowers [9]. The milky sap of stem and leaf is applied to skin diseases such as herpes, scabies, and ulcers [10]. Moreover its bark is bruised and applied as plaster over hard tumours [11], whereas the latter taxon finds use as being purgative, cardiotonic, diuretic, and hypotensive [12]. Leaves are useful in inflammation, rheumatism, bronchitis, cholera, cold, and cough and also useful as being antipyretic, antifungal, stimulant, and so forth [13]. The aerial parts of plant are reported to contain steroids, flavonoids, and alkaloids [14]. The root bark of* Plumeria alba* shows the presence of iridoids, tannins, and alkaloids [15]. The plant is reported to contain mixture of amyrins, *β* sitosterol, scopotein, iridoids, isoplumericin, plumeride, plumeride coumerate, and plumeride coumerate glucoside [16, 17].

As far as we know, there have been no reports on the antiarthritic activity of this plant in animals so far. In this study fractions of hydroalcoholic extract of* Plumeria alba* were evaluated in animals to substantiate and expand its clinical applications.

## 2. Materials and Methods

### 2.1. Plant Material Collection

The leaves of* Plumeria alba* were collected from Chaudhary Devi Lal Park, in Yamuna Nagar, Haryana, India, February 2011, and identified by Dr. H. B. Singh, Head of Raw Materials Herbarium and Museum (RHMD), New Delhi. A voucher specimen of the plant (reference no. NISCAIR/RHMD/CONSULT/2012-13/2047/55) has been deposited at the herbarium for future reference.

### 2.2. Preparation of Extract

The air-dried leaves (2 kg) were thoroughly washed under running tap water so as to remove any type of contamination, air-dried in shade, powdered in grinder, and passed through sieve of mesh size of no-40. The extraction was done with aqueous ethanol (30 : 70) mixture by hot Soxhlet extraction method and the extract was concentrated in a rotary evaporator under reduced pressure, yielding 720 g (36%) of crude hydroalcoholic extract (HAPA). The dried crude extract was preserved in airtight glass container at 4°C–8°C.

### 2.3. Bioguided Fractionation

Initially, HAPA was partition-fractionated with 1 : 1 (v/v, volume ratio) of n-hexane and ethanol (50%); the mixture was shaken vigorously and kept for about 30 min to make the two layers separate. The upper layer consisting of n-hexane was removed and concentrated in a rotary evaporator to obtain n-hexane extract (HPA). The same procedure was repeated with the bottom layer by using equivalent volume of other solvents like dichloromethane, ethyl acetate, n-butanol, ethanol, and water to obtain DCMPA, EAPA, BPA, EPA, and APA, respectively.

### 2.4. Preliminary Phytochemical Screening

Phytochemical analysis was performed using standard procedures to identify chemical constituents as described by Khandelwal [18]. Precoated thin-layer chromatography plates of silica gel 60 F254 + 366 with support of aluminium sheets 0.1 mm thick and 10 cm × 10 cm were used for the preliminary chromatographic characterization of fractions. All the fractions (10 mg) were dissolved in methanol (mL). The mobile phase used was chloroform: methanol = 7 : 3. The plates were sprayed with natural products reagent and polyethylene glycol (NP/PEG) for detecting flavonoids. Phenolic compounds are detected after exposing the plates to ammonia vapours and immediate observation of fluorescent spots under UV light [19]. Anisaldehyde in sulphuric acid was used as detecting agent for steroids.

### 2.5. Analysis of the Extract

Stigmasterol and extracts were accurately weighed and dissolved in methanol to produce a solution containing 1.90 mg/25 mL. The sample was run for 20.00 min, and volume of 5.00 *μ*L was injected into the HPLC instrument (MAKE). HPLC analysis was performed on a Hypersil C 18 150 × 4.6 mm (DRDC/AD/36) column (MAKE) with a UV detector. The mobile phase consisted of methanol : acetonitrile : isopropyl alcohol: 40 : 20 : 20. The flow rate was 1.0 mL/min and the detection wavelength of the UV detector was set at 228 nm. The column temperature was set at 30°C.

### 2.6. Drugs and Chemicals

Formaldehyde (S.d. Fine Chemicals Ltd., Mumbai, India), FCA (Difco Laboratories, USA), and diclofenac (Symed Pharmaceutical Pvt., Ltd., Hyderabad). Solvents used for extraction and chemicals used for phytochemical analysis were of analytical grade procured from approved organization.

### 2.7. Dose and Route of Administration

The dosages of EAPA and BPA used in this study were 100 and 200 mg/kg per day, which are the optimal dosages ascertained in our previous study [20]. Fresh drug solutions were prepared in Tween 80, (2% v/v) at the time of administration, and were administered perorally (p.o.) so as to avoid any additional stress to the animals.

### 2.8. Preparation of Reference Drug

The reference anti-inflammatory drug diclofenac was dissolved in normal saline for the study. The drug solution was freshly prepared and administered orally at dose 4 mg/kg in volumes not exceeding 10 mL/kg.

### 2.9. Animals

Healthy Sprague-Dawley rats (200–300 g) of either sex, purchased from a disease free animal house of the National Institute of Pharmaceutical Sciences and Education Research, Mohali, Punjab, India, were housed in the animal house, Institute of Pharmaceutical Sciences, Kurukshetra University, Kurukshetra, Haryana, India. The animals were fed with commercially available feed and were maintained under standard conditions of temperature (25°C ± 5°C), relative humidity (55 ± 10%), and 12/12 h light/dark cycle. They were transferred to the laboratory twelve hours prior to the experiments and given only water ad libitum. In all the experiments, the animals were kept in cages with raised floors of wide mesh, to prevent coprophagy. The animals were housed and cared for in accordance with the Federal Government Legislation on Animal Care. The experiments were also authorized by the Institutional Animals Ethical Committee (IAEC) for Animal Care (register number: 562/GO/02/a/CPCSEA) and were in accordance with Committee for the Purpose of Control and Supervision of Experiments on Animals (CPCSEA) Guidelines, India.

### 2.10. Experimental Procedure

#### 2.10.1. Acute Nonimmunological Formaldehyde-Induced Arthritis in Rats

Sprague-Dawley rats were divided into five groups of six animals each. Baseline recording of the paw volume was made by using plethysmometer (Ugo Basile 7140, Italy) [21]. Group I (vehicle control) received Tween 80, (2% v/v, p.o.), group II received formaldehyde, groups III and IV received EAPA and BPA (100 and 200 mg/kg, p.o.), respectively, and animals of group V received diclofenac sodium (4 mg/kg, p.o.) for 10 days. On day 1, 30 min after the drug administration, acute nonimmunological arthritis was induced by subplantar injection of 0.1 mL formaldehyde (2% v/v) into the right hind paw of all the animals except group I animals and repeated on day 3. Arthritis was assessed by measuring the mean increase in paw volume over a period of 10 days.

#### 2.10.2. Chronic Immunological CFA-Induced Arthritis in Rats

*(a) Induction of Arthritis*. Adjuvant arthritis was induced in rats according to the method described by Newbould, 1963, with slight modification [22]. In this process, the initial hind paw volumes (both left and right) of the experimental animals were measured by water displacement plethysmography. Following anaesthesia with ketamine (120 mg/kg), 0.1 mL of FCA (a suspension of heat-killed* Mycobacterium tuberculosis* in mineral oil) was then injected into the subplantar tissue of the right posterior paw. The volume of the injected (ipsilateral), primary response and uninjected (contralateral), secondary response hind paws was measured on alternate days from 2 to 21 days after the adjuvant injection. Unilateral inflammatory edema of the ipsilateral paw peaking at around days 6–8 was indicative of successful induction of adjuvant arthritis.

*(b) Experimental Design.* Grouping of animals is the same as prescribed in formaldehyde-induced nonimmunological arthritic model.

 Group I received Tween 80 (2% v/v, p.o.), vehicle control.
 Group II received Tween 80 (2% v/v, p.o.), FCA control.
 Group III received EAPA (100 mg/kg, p.o.).
 Group IV received BPA (200 mg/kg, p.o.).
 Group V received diclofenac sodium (4 mg/kg, p.o.).

Thirty minutes after administration of vehicle/drug, arthritis was induced by subplantar injection of FCA. This was designated as day 0. After immunization with FCA, all groups were maintained on vehicle/drug treatment for 20 more days [23]. Antiarthritic activity of active fractions was evaluated on paw volume, arthritic score, pain withdrawal latency, and fall-off time on days 0, 2, 4, 6, 8, 10, 12, 14, 16, 18, and 21. Moreover body weights of animals were monitored regularly during the course of the experiment. On day 21, blood was withdrawn by retroorbital puncture for assessment of haematological parameters and animals were sacrificed under light ether anaesthesia to study histopathology of joints.

### 2.11. Parameters Assessment

#### 2.11.1. Arthritic Score

Photographs of the arthritic rats were taken on day 21 with a digital 10-megapixel (Panasonic DMC-FS42) camera. Inflammation of each paw was graded blindly by the same person for all rats on day 21 according to the extent of erythema and oedema of the joints, using the criteria [24, 25] as follows: normal paw = 0, mild swelling and erythema of digits = 1, moderate swelling and erythema of digits = 2, severe swelling and erythema of digits = 3, and gross deformity and inability to use limbs = 4. The arthritic score for each rat on day 0 was determined to be 0. The scores for each paw were then added to get the total arthritic score. Thus, the maximum possible score for each animal was 16.

#### 2.11.2. Paw Volume

The paw volume of both hind paws was measured just before FCA injection on day 0 and on alternate days after 2 to 21 days using a plethysmometer.

#### 2.11.3. Motor Incoordination Test

Motor incoordination was evaluated by Rotarod apparatus as described earlier [26]. Rats were placed on the rotating rod of device for 1 min. The time taken for the falling of rats from the roller, during the period of 1 min, was recorded.

#### 2.11.4. Antinociceptive Activity

The apparatus consists of a hot plate on which the rats were placed for testing (Eddy's hot plate method) [27]. Pain threshold was determined by the latency for nociceptive response (withdrawal of any paw) with a maximum cut-off time of 15 sec for all groups.

#### 2.11.5. Measurement of Organs Weight

The rats were sacrificed with ether on the 21st day, the spleen and thymus were removed, and the weight of the organs was recorded and corrected for 100 g body weight.

#### 2.11.6. Haematological Assessment

On the 21st day, blood was withdrawn from each animal through the retroorbital plexus into a test tube containing anticoagulant (5% EDTA) and haematological parameters were determined. The red blood cells (RBC) were determined by the method of Huxtable [28]; white blood cell count (WBC) was determined using Raghuramulu [29]. Haemoglobin concentration was estimated by the cyanmethemoglobin method of Drabkin and Austin [30]. Erythrocyte sedimentation rate (ESR) was determined using the Wintrobe method [31].

### 2.12. Histological Analysis

After sacrifice on the 21st day, ankle joints were removed from the hind paw, weighed, and fixed for 24 h in 10% formalin. After decalcification in 5% formic acid, processed for paraffin embedding, tissue sections (5 *μ*m thick) were stained with haematoxylin and eosin. An experienced pathologist (Dr. Neeraj Mittal), unaware of the different drug treatment, evaluated the slides under light microscope for the presence of hyperplasia of synovium, inflammatory cells, fibrosis, and destruction of joint space.

### 2.13. Statistical Analysis

The results were expressed as mean ± SEM. Statistical comparison was made between drug-treated group and arthritic-control group. Statistical difference between two means was determined by one-way ANOVA followed by Dunnett's multiple comparison test using Instat 3 statistical computer software. Only those mean values showing statistical difference *P* < 0.01 or *P* < 0.05 were considered as statistically significant.

## 3. Results

### 3.1. Preliminary Phytochemical Screening

**Figure 1:**
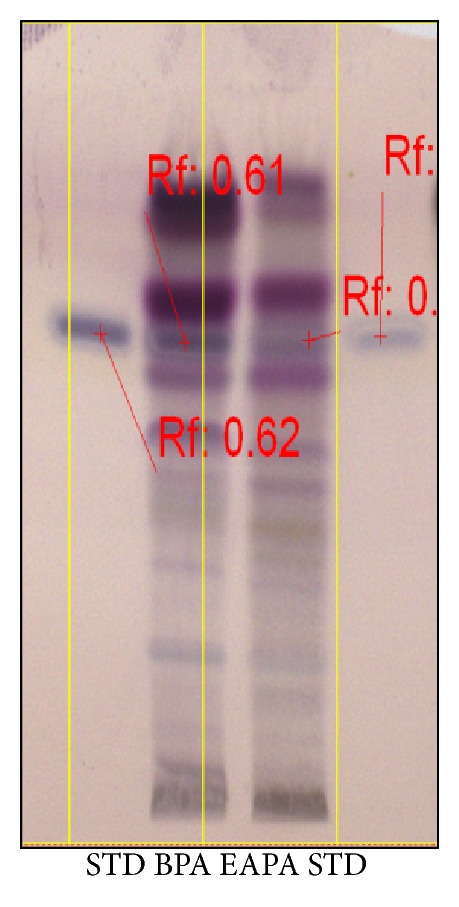
HPTLC fingerprinting of stigmasterol, BPA, and EAPA showing the Rf value.

**Figure 2:**
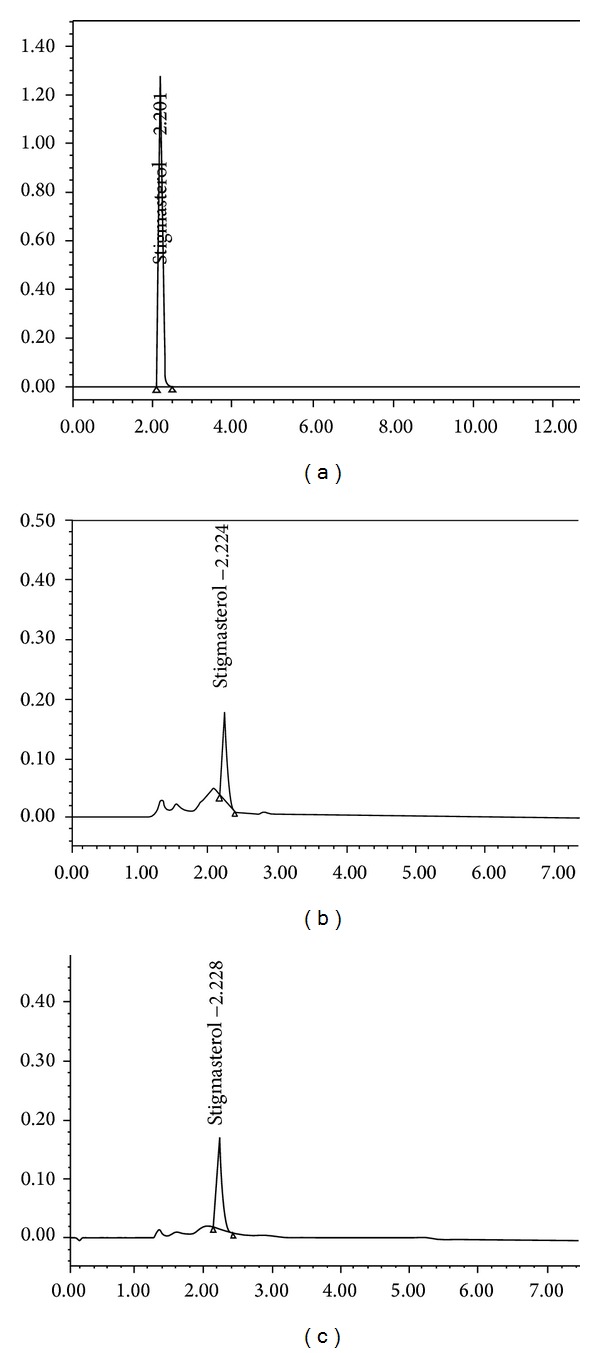
HPLC fingerprinting of stigmasterol (a), EAPA (b), and BPA (c) showing the presence of stigmasterol in the extract.

Preliminary phytochemical screening of EAPA and BPA revealed the presence of alkaloids, glycosides, terpenoids, flavonoids, saponins, and steroids. In addition, tannins were also present in BPA. HPTLC and HPLC studies confirmed the presence of stigmasterol (0.052 in EAPA and 0.0760 in BPA) in the fractions (Figures 1 and 2).

### 3.2. Formaldehyde-Induced Paw Edema

**Figure 3:**
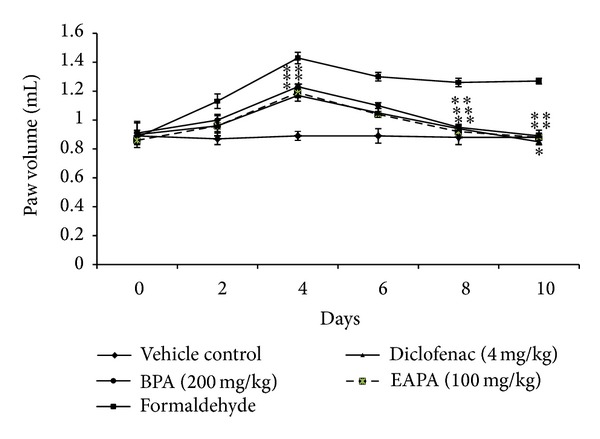
Effects of EAPA and BPA on formaldehyde-induced paw edema. Values are plotted as the mean ± SEM, *n* = 6, in each group; significant reduction in paw volume was analysed by one-way analysis of variance followed by Dunnett's multiple comparisons test using GraphPad Instat software; *P < 0.05 and **P < 0.01 compared to formaldehyde control.

Administration of 2 per cent formaldehyde on days 1 and 3 showed ankle joint swelling in the injected limb of all the animals. The joint swelling increased up to 4 days and then starts decreasing. EAPA and BPA significantly suppressed the joint swelling when compared with arthritic control between day 4 and day 10 after formaldehyde treatments (Figure 3). Even though the maximum inhibition of paw volume was produced by diclofenac sodium, there was only marginal difference in the efficacies of standard drug and both fractions of the extract.

### 3.3. FCA Induced Arthritis

**Figure 4:**
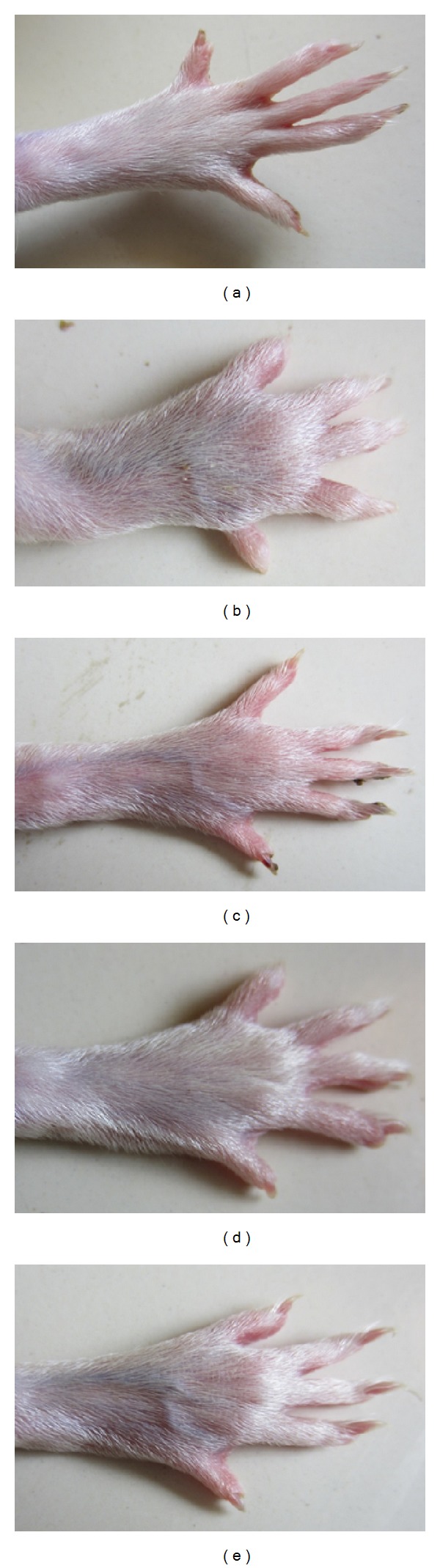
Morphological representations of rat paw after subplantar administration of FCA. (a) Vehicle control; (b) FCA treated rats; (c) EAPA (100 mg/kg); (d) BPA (200 mg/kg); (e) diclofenac (4 mg/kg) treated rats.

Freund's complete adjuvant in rats induced significant inflammatory response characterized by paw swelling in both injected (ipsilateral) and noninjected (contralateral) paws. The response was biphasic, consisting of acute and polyarthritis phase. The acute manifestation of disease was corresponding to days 0–8 characterized by inflammation of ipsilateral paw, followed by polyarthritis phase corresponding to 9–21 days, characterized by involvement of contralateral paw (Figure 4).

#### 3.3.1. Effect of EAPA and BPA on Arthritic Index

**Figure 5:**
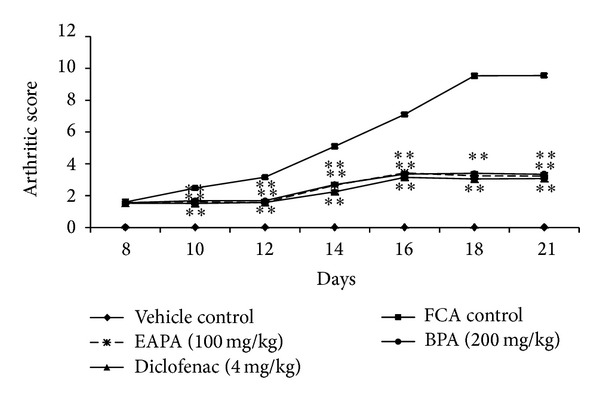
Effects of EAPA and BPA on arthritic score in FCA model. Values are plotted as the mean ± SEM, *n* = 6, in each group; decreased arthritis score was analysed by one-way analysis of variance followed by Dunnett's multiple comparisons test using GraphPad Instat software; *P < 0.05 and **P < 0.01 compared to FCA control.

In the CFA-induced arthritis control group, the adjuvant ipsilateral paw joints started to show swelling and rigidity around 8–10 days and arthritic score reached the maximum level on day 18. All the treated groups suppressed the arthritic score significantly from day 10 to day 21. There were significant reductions in arthritic indices (*P* < 0.01) for both fractions EAPA and BPA (100 and 200 mg/kg), respectively, as well as reference drug compared with the FCA-arthritic animals (Figure 5). The results of both extracts were comparable with standard drug ibuprofen (4 mg/kg).

#### 3.3.2. Effect of EAPA and BPA on Body Weight

**Figure 6:**
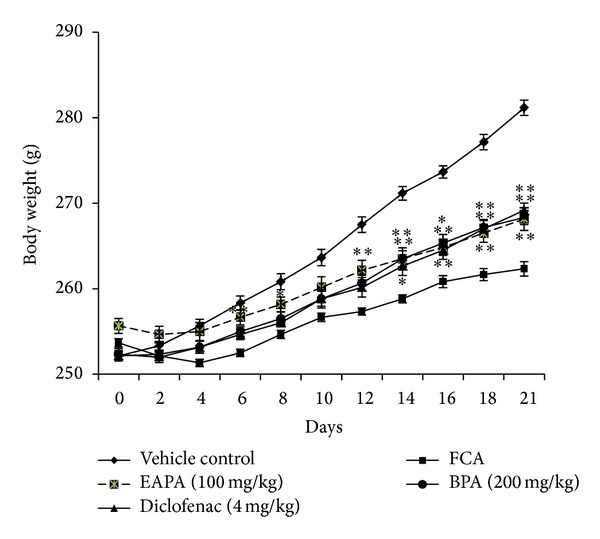
Effects of EAPA and BPA on body weight in FCA model. Values are plotted as the mean ± SEM, *n* = 6, in each group; significant reduction in body weight was analysed by one-way analysis of variance followed by Dunnett's multiple comparisons test using GraphPad Instat software; *P < 0.05 and **P < 0.01 compared to FCA control.

Animals in which arthritis has been induced gained less weight due to generation of immune responses which was lower than vehicle controls, throughout the study. But treatment with fractions EAPA and BPA, (100 and 200 mg/kg), respectively, showed significant increase (*P* < 0.05, *P* < 0.01) in body weight from day 1 to day 21 as compared to arthritic-control rats. Rats treated with diclofenac (4 mg/kg) also showed significant improvement in body weight throughout the study as compared to arthritic-control animals (Figure 6).

#### 3.3.3. Effect of EAPA and BPA on Paw Volume

**Figure 7:**
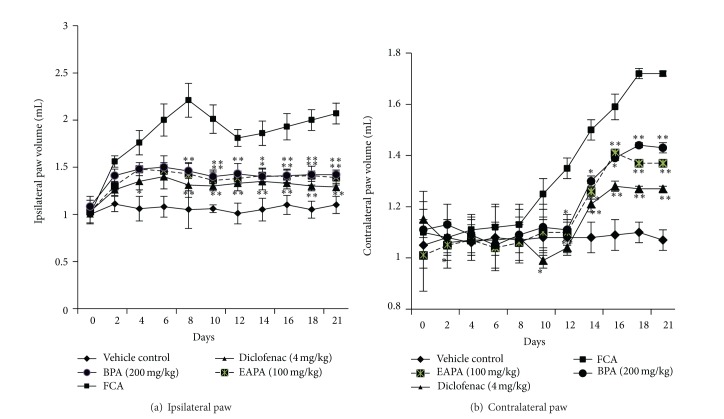
Effects of EAPA and BPA on (a) ipsilateral paw and (b) contralateral paw. Values are plotted as the mean ± SEM, *n* = 6, in each group; significant reduction in paw volume was analysed by one-way analysis of variance followed by Dunnett's multiple comparisons test using GraphPad Instat software; *P < 0.05 and **P < 0.01 compared to FCA control.

The time dependent changes in the hind paw volumes of rats are shown in Figures 7(a) and 7(b). The volume of the ipsilateral (primary lesions) paw as well as contralateral (secondary lesions) paw in the FCA rats increased progressively. This also had shown a biphasic response where there was a small decrease in paw volume from day 8 to 10. However, this was nonsignificant change. After the 12th day progressive increase in paw volume was seen in all FCA administered groups. The rats receiving diclofenac or fractions of extracts (EAPA and BPA, 100 and 200 mg/kg) significantly (*P* < 0.05, *P* < 0.01) decreased the primary lesions and secondary lesions seen after 10 days of study, when compared to arthritic control. Normal animals kept under experiment did not show paw swelling.

#### 3.3.4. Effect of EAPA and BPA on Fall-Off Time

**Figure 8:**
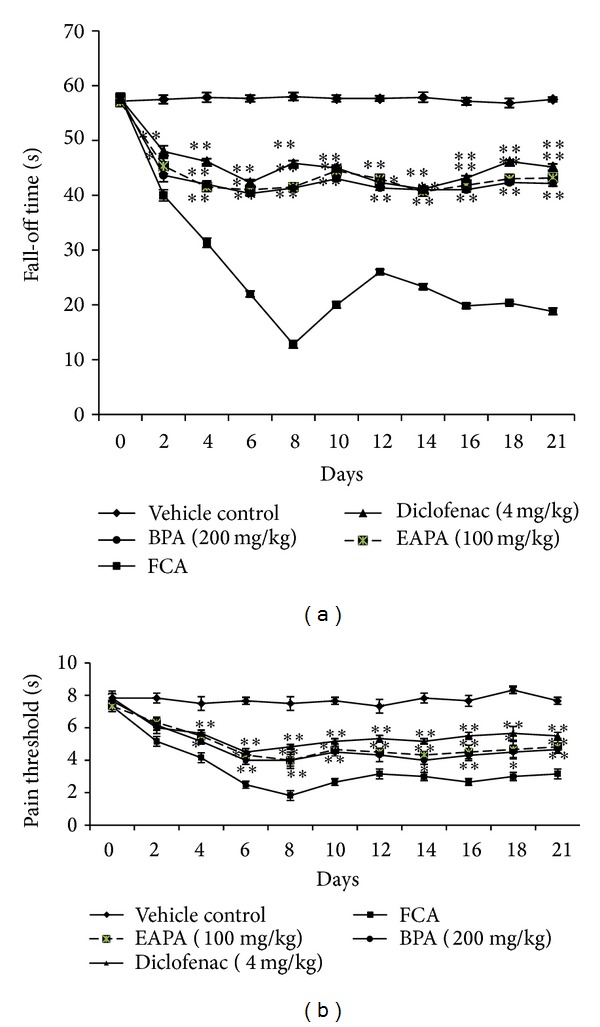
Effects of EAPA and BPA on (a) fall-off time (s) and (b) pain threshold (s). Values are plotted as the mean ± SEM, *n* = 6, in each group; significant reduction in fall-off time and pain threshold were analysed by one-way analysis of variance followed by Dunnett's multiple comparisons test using GraphPad Instat software; *P < 0.05 and **P < 0.01 compared to FCA control.

Rotarod method was used to evaluate motor incoordination by determining the mean fall-off time. When compared to vehicle control group, FCA treatment group showed consistent decrease in fall-off time from day 0 to 8 and then showed improvement up to day 14 and consistent fall-off time thereafter. With treatment of extracts (EAPA and BPA, 100 and 200 mg/kg) and diclofenac (4 mg/kg), initially there was decrease in fall-off time from day 2 to 6; then increase in fall-off time was observed up to 12 days; after that, consistent fall-off time was observed throughout the study (Figure 8(a)).

#### 3.3.5. Effect of EAPA and BPA on Nociceptive Threshold

There was consistent decrease in paw withdrawal latency in FCA treated rats throughout the study as compared to normal rats after subplantar FCA administration. When compared with FCA treated rats, EAPA (100 mg/kg), BPA (200 mg/kg), and diclofenac (4 mg/kg) showed decrease in paw withdrawal latency from day 2 to 8; then significant (*P* < 0.05, *P* < 0.01) improved paw withdrawal latency was observed which was maintained till the end of study (Figure 8(b)).

#### 3.3.6. Effect of EAPA and BPA on Organs Weight

In the experiment, the mean spleen weight was increased whereas there was decrease in thymus weight of the FCA treated rats as compared to vehicle control group (Table 1). It was suggested that splenomegaly was apparent. Rise in spleen weight was significantly (*P* < 0.01) inhibited in rats treated with EAPA (100 mg/kg) and BPA (200 mg/kg) as compared to FCA treated rats. However, only treatment with EAPA and diclofenac attenuated the decreased weight of thymus, significantly (*P* < 0.05 and *P* < 0.01), respectively.

#### 3.3.7. Effect of EAPA and BPA on Haematological Parameters

Haematological assessment indicated no significant difference (*P* > 0.05) in red blood cells and white blood cell counts of EAPA (100 mg/kg), BPA (200 mg/kg), and diclofenac (4 mg/kg) treated groups compared to the FCA-arthritic animals (Table 2). Erythrocyte sedimentation rate was significantly (*P* < 0.01) low with both fractions and standard reference drug as compared to FCA-arthritic rats. Further haematological studies revealed significantly (*P* < 0.01) more hemoglobin level only in animals treated with diclofenac (4 mg/kg).

#### 3.3.8. Effect of EAPA and BPA on Histopathological Studies

**Figure 9:**
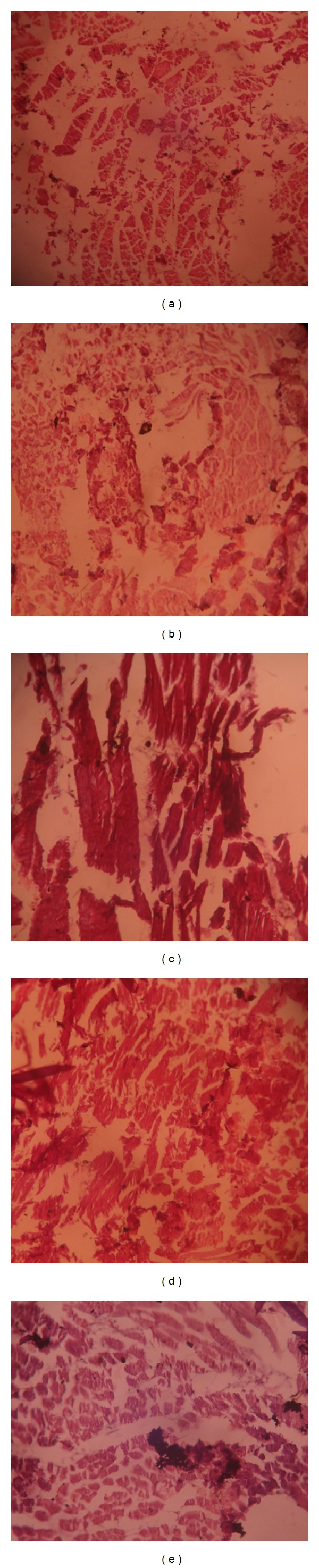
Histology of the arthritis developing 21 days after immunization with FCA compared with unimmunized Sprague-Dawley rats. (a) Vehicle control; (b) FCA control; (c) EAPA (100 mg/kg); (d) BPA (200 mg/kg); (e) diclofenac (4 mg/kg). Magnification ×100; thickness: 5 *μ*.

Histopathological studies of the tibiotarsal joints show destructive lesions in connective tissue, vascularity into joint space, and granuloma formation in the FCA treated animals (Figure 9). There was present normal connective tissue structure with absence of necrosis in the tibiotarsal joint of vehicle control group. Diclofenac treatment showed normal connective tissue of tibiotarsal joint with the presence of lesser edema and absence of necrosis. EAPA and BPA treated rats produced knee joints protection compared to arthritic rats by reducing the inflammation and necrosis.

## 4. Discussion

Various animal models are used in the preclinical research in inflammatory arthritis [32, 33]. Formaldehyde-induced paw edema and Freund's complete adjuvant induced arthritis are well established rat model which have been extensively used in the evaluation of antiarthritic potential of various agents in preclinical research [34–36]. The present study evaluated the antiarthritic activity of ethyl acetate and n-butanol fraction of hydroalcoholic extract of* P. alba*. Dosage selection for fractions of plant (ethyl acetate, 100 mg/kg and n-butanol, 200 mg/kg) was based on the previous studies conducted by our research group [20]. The antiarthritic potential of EAPA and BPA was initially investigated against formaldehyde- induced paw edema, which is one of the most common methods for screening of antiarthritic agents [37]. The localized inflammation, induced by injection of formaldehyde into rat paw, is biphasic in nature [38]. In the present study, EAPA and BPA decreased the paw edema. It might be due to the certain alterations in the inflammatory response comparable with standard drug diclofenac with possible antiarthritic potential.

The standard drug diclofenac sodium prevented the adjuvant induced arthritis which is consistent with previous studies [39–41]. The diclofenac, a nonsteroidal anti-inflammatory drug, was used for comparison because it is commonly prescribed for the treatment of arthritis and acts mainly through the inhibition of cyclooxygenase and prostaglandin production [42]. The antiarthritic activity of EAPA and BPA was further confirmed in FCA induced arthritis as it shows several clinical and immunological similarities to human RA [3]. The development of FCA induced arthritis in the rat can be characterized by pronounced swelling in the hind paw primary chronic arthritis [22]. In primary reaction, prostaglandin generations occur, which is followed by swelling in contralateral and front paws (secondary chronic arthritis) in which autoantibodies are generated [36, 43]. A good antiarthritic agent should be able to suppress one or both of these phases. Recent studies have shown the role of various proinflammatory mediators such as tumour necrosis factor (TNF-*α*), interleukin-1 beta (IL-1*β*), and platelet derived growth factor (PDGF) in the pathogenesis of RA [44, 45]. Treatment with EAPA and BPA significantly inhibits the progression of the rheumatoid arthritis via decreasing the paw thickness. The effect of fractions may be due to inhibition of release of inflammatory mediators, indicating its anti-inflammatory effect.

The severity of arthritis was expressed as arthritic score is the index of joint inflammation. Arthritic score is the sum of four paws after immunization and values before immunization [31]. Arthritic score of EAPA and BPA treated groups was significantly lower than FCA treated rats, thus distinguishing the immunosuppressive effects of fractions from its anti-inflammatory effect. Progression of disease status and response to anti-inflammatory therapy are indirectly linked with change in body weight as RA is associated with weight loss and loss of lean body mass, known as rheumatoid cachexia [46, 47]. Earlier observations by Roubenoff et al. suggest the decreased physical activity, muscle strength, and decreased daily performance [48, 49]. It was observed that intestinal absorption of ^14^C-glucose and ^14^C-leucine was reduced in inflammation whereas anti-inflammatory drug treatment has improved the decreased absorption capacity [50, 51]. In the present investigation, the FCA treated rats showed less body weight gain as compared with diclofenac and extract treated arthritic rats. Thus, it can be concluded that increased body weight may be due to the restoration of the absorption capacity of intestine that shows effective management of rheumatoid cachexia.

Several studies were conducted to evaluate hyperalgesia by using arthritic rats. Eddy's hot plate is a quantitative method for determination of hyperalgesia related to behaviours [26, 52]. The change, observed after acute pain stimulus in the nonaffected limb, shows the involvement of inhibiting controls caused by obvious long standing nociceptive input from the contralateral arthritic limb. It has been observed by previous researchers that increased pain sensitivity is linked with decreased hyperalgesia latency [26]. In the present investigation we observed that EAPA and BPA showed significant improvement in nociceptive threshold.

Spleen is a vital organ serving as the available source of cells and antibody formation, known to be involved in immunological response in adjuvant arthritis [53]. In the spleen of arthritic rats, increased cellularity is observed. Due to splenomegaly, increase in the weight of spleen occurs [54, 55]. EAPA and BPA attenuated the increased weight of spleen and decreased weight of thymus induced by subplantar administration of FCA, probably by suppression of splenic lymphocytes and subsequent inhibition of the infiltration lymphocytes into synovium, which may be due to the immunostimulant effect of EAPA and BPA.

The significant low level of ESR in the EAPA, BPA, and diclofenac treated arthritis rats indicates anti-inflammatory potential. The ESR is indirect method for the measurement of inflammation in the body. Erythrocytes move closer, stack up in a group, due to protein production in inflammation, become denser, and settle faster that increases the erythrocyte sedimentation rate [56]. In various stress conditions, cell necrosis, and inflammation, ESR is elevated [57].

Histopathological studies of the paws contribute towards the treatment of arthritis with EAPA and BPA despite evidence of pathology in the arthritic animals treated with the reference drug.

The antiarthritic effect of fractions (EAPA and BPA) of hydroalcoholic extract of* P. alba *could be due to the presence of flavonoids, alkaloids, terpenoids, glycosides, saponins, tannins, and steroids detected after preliminary phytochemical screening of the fractions. Nonspecific antiarthritic activity may be due to the combined effect of the different phytoconstituents present.

## 5. Conclusion

The result of the present study suggests that antiarthritic potential of EAPA and BPA may be due to the protection of synovial membrane, vascular permeability, and prevention of cartilage destruction, thereby improving the health status through haematinic properties. The effect may be due to the inhibition of phospholipase A_2_ and prostaglandins due to similar effect of diclofenac.

It also demonstrates its beneficial effect during recovery from arthritis by including Hb, ESR, and body weight along with clinical signs including paw oedema, thermal hyperalgesia, and histopathological examination. The study established that the ethyl acetate and n-butanol fractions of* P. alba* possess antiarthritic activity in Sprague-Dawley rats. The present study predicts that the* P. Alba* provides pharmacological rationale for the traditional use of the plant against inflammation disorders like rheumatoid arthritis.

However, further studies are needed to identify and isolate the possible phytoconstituent(s) involved in the antiarthritic activity, which would facilitate the use of* P. alba* in inflammatory disease.

## Figures and Tables

**Table 1:** Effect of EAPA and BPA treatments on thymus and spleen weight.

Treatment	Thymus weight (mg/100 g b.wt.)	Spleen weight (mg/100 g b.wt.)
Vehicle control	103.5 ± 2.01	185.5 ± 4.09
FCA	81.00 ± 0.81	249.16 ± 2.71
EAPA (100 mg/kg)	86.16 ± 0.94*	214.83 ± 1.77**
BPA (200 mg/kg)	83.83 ± 0.98	215.5 ± 1.78**
Diclofenac (4 mg/kg)	91 ± 0.96**	209.83 ± 4.20**

Values are plotted as the mean ± SEM, *n* = 6, in each group; significant reduction was analysed by one-way analysis of variance followed by Dunnett's multiple comparisons test using GraphPad Instat software; *P < 0.05 and **P < 0.01 compared to FCA control.

**Table 2:** Effect of EAPA and BPA on haematological profile.

Treatment	RBC (×10^6^/*μ*L)	WBC (×10^3^/*μ*L)	Hb (g/dL)	ESR (mm)
Vehicle control	8.05 ± 0.12	11.48 ± 0.16	13.21 ± 0.10	3.05 ± 0.07
FCA	7.58 ± 0.11	14.15 ± 0.18	11.56 ± 0.18	9.43 ± 0.12
EAPA (100 mg/kg)	8.00 ± 0.09	12.63 ± 0.87	12.48 ± 0.42	3.83 ± 0.10**
BPA (200 mg/kg)	7.98 ± 0.14	12.35 ± 0.61	12.48 ± 0.27	3.96 ± 0.07**
Diclofenac (4 mg/kg)	8.00 ± 0.12	12.21 ± 0.43	13.08 ± 0.09**	3.53 ± 0.10**

Values are plotted as the mean ± SEM, *n* = 6, in each group; significant reduction was analysed by one-way analysis of variance followed by Dunnett's multiple comparisons test using GraphPad Instat software; **P < 0.01 compared to FCA control.
